# Supplementary material for: Consecutive Injection of High-Dose Lipopolysaccharide Modulates Microglia Polarization via TREM2 to Alter Status of Septic Mice
Source: Brain Sci. 2023 Jan 11;13(1):126. doi: 10.3390/brainsci13010126 (PMC9856382; doi:10.3390/brainsci13010126)
Supplement: Supplementary file 1 [file brainsci-13-00126-s001.zip › brainsci-2116987-supplementary.pdf]

Fig. S1

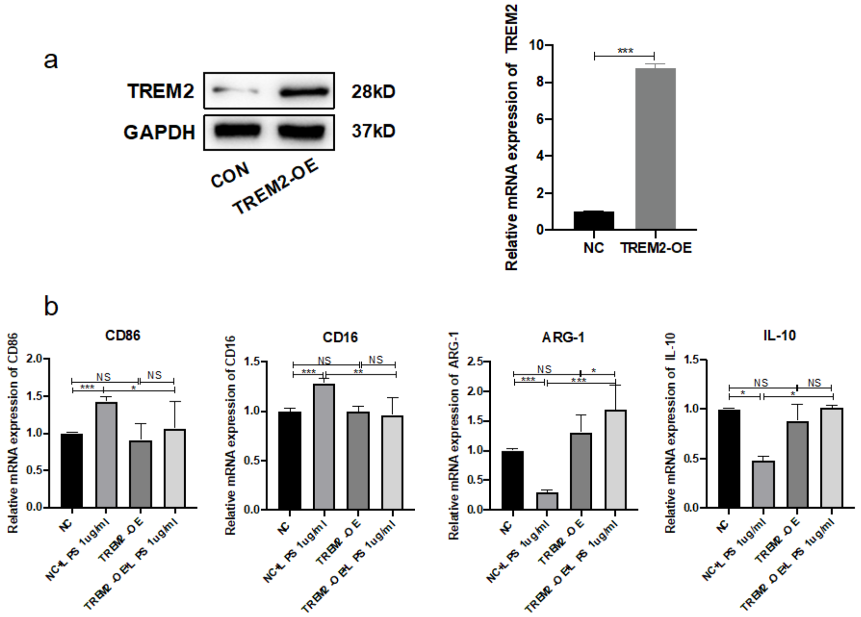

Figure S1 Polarization of microglia overexpressing TREM2 after LPS treatment

A. Protein and mRNA Expression of in TREM2-OE BV-2 cell.

B. mRNA expression of M1-type microglia markers (CD16 and CD86) and M2-type microglia markers (Arg-1 and IL-10) after 24h treatment of TREM2-OE and NC cells with different concentrations of LPS by PCR.

Fig. S2

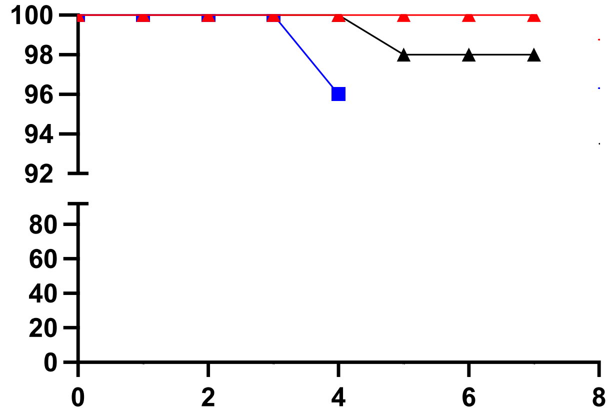

Figure S2. Survival of each group of mice

Fig. S3

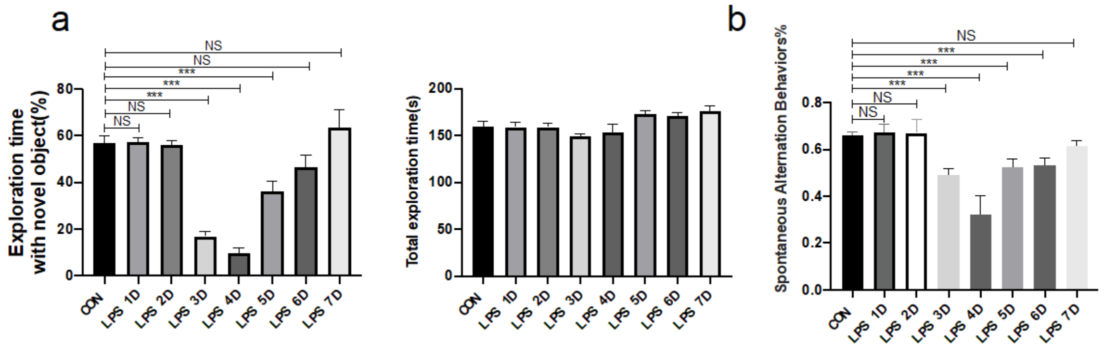

Figure. S3 Behavior of each group of mice

A. Total and percentage of detection time of new objects in each group of mice.

B. Percentage of mice in each group entering the different arms of the Y-maze in sequence.
